# Prediction of neoadjuvant therapy efficacy in gastric cancer: the interplay between biomarkers and radiomics and its potential for clinical translation

**DOI:** 10.3389/fonc.2025.1642398

**Published:** 2026-01-15

**Authors:** Zhou Yufeng, Xu Le, Chen Gong, Lin Dandan

**Affiliations:** Cancer Center, Renmin Hospital of Wuhan University, Wuhan University, Wuhan, China

**Keywords:** gastric cancer, AI, neoadjuvant therapy, biomarkers, prognosis model, radiomics

## Abstract

Neoadjuvant therapy (NACT) for locally advanced gastric cancer (LAGC) plays a crucial role in improving surgical resection rates and patient prognosis. However, there is significant heterogeneity in patient responses to treatment, necessitating effective predictive tools for personalized therapy. This review systematically summarizes the latest research advancements in biomarkers and imaging models for predicting the efficacy of neoadjuvant treatment in gastric cancer. In the field of biomarkers, systemic immune-inflammation index (SII), microRNAs (miRNAs), and aspartate β-hydroxylase (ASPH) are molecular markers that influence chemotherapy sensitivity by modulating the tumor microenvironment or signaling pathways. Among them, SII, a low-cost and non-invasive inflammatory marker, has been shown to predict patient survival and treatment response. Differential expression of miRNAs (e.g., miR-7, miR-143) provides molecular evidence for evaluating the efficacy of neoadjuvant chemotherapy. ASPH, on the other hand, promotes chemotherapy resistance by activating the Notch/SRC pathway, making it a potential therapeutic target. Additionally, immune checkpoint inhibitors (ICIs) combined therapy has demonstrated a high pathological complete response rate in patients with high PD-L1 expression or the dMMR/MSI-H subtype. Clinical trials of Claudin 18.2-targeted therapies (e.g., Zolbetuximab) further expand personalized treatment options. Radiomics and deep learning models (e.g., DLDRN, DLCS), by integrating clinical data with radiological features, offer non-invasive methods to predict tumor response and survival risk, providing valuable support for clinical decision-making. This review aims to systematically collate the latest evidence on biomarkers and radiomics for predicting the efficacy of neoadjuvant therapy in gastric cancer. To achieve this objective, we focus on three core domains: (1) key biomarkers with clinical translational potential (such as SII, miRNA, PD-L1, etc.); (2) CT- and MRI-based radiomics predictive models; (3) Future prospects for multi-omics integration strategies. Despite the abundance of research in this field, this paper prioritizes the analysis and discussion of prospective or high-quality retrospective studies that include explicit efficacy prediction endpoints (such as pCR, TRG, AUC) to ensure the reliability of the evidence presented. This review emphasizes that multi-omics integrated predictive models and the clinical translation of targeted therapies represent critical directions for future research, aiming to optimize the neoadjuvant treatment strategies for locally advanced gastric cancer.

## 1 Introduction

Gastric cancer (GC) ranks among the leading causes of cancer incidence and mortality worldwide (1, 2), with patients suffering from locally advanced gastric cancer (LAGC) facing a poor clinical prognosis due to the aggressive nature of the tumor and a high risk of postoperative recurrence. Neoadjuvant chemotherapy (NACT), which reduces tumor size and downstages the disease, has emerged as a feasible treatment option for LAGC (3–6) and is recommended in the guidelines of the Chinese Society of Clinical Oncology (CSCO) (7). However, there is significant interindividual variability in the clinical efficacy of NACT, and a lack of treatment sensitivity in some patients may lead to tumor progression or missed surgical opportunities.

Current treatment protocols for NACT in GC lack standardization and are primarily guided by clinical trials. Early studies focused on chemotherapy regimens and their efficacy in achieving clinical resectability as the primary endpoint. With the advent of immunotherapy, neoadjuvant protocols have increasingly incorporated immune checkpoint inhibitors, prompting the exploration of novel biomarkers to predict treatment response and prognosis. This evolution underscores the shift toward personalized medicine, leveraging multimodal approaches to optimize patient outcomes. (**Table 1**)

**Table 1:** Summary of neoadjuvant clinical studies in gastric cancer.

Study or principal investigator	Phase	Cell of origin	Treatment arms	Number (n)	Primary endpoints	Key findings	Reference
OEO2	III	EC/GEJ	FP programme (two cycles) + surgeries vs surgeries	802	OS、DFS、R0 resection rate	5-yearOS: 23.0% vs.17.1% (HR = 0.84, p=0.03);R0 resection rate:79.3%vs. 70.3% (p=0.03)	(8)
EORTC 40954	III	GEJ	CF programme(two cycles) + surgeries vs surgeries	144	OS、DFS#x3001;R0 resection rate	R0 resection rate: 81.9% vs. 66.7% (p=0.036)	(9)
NeoFLOT	II	GEJ or gastric adenocarcinoma	FLOT programme (six cycles) + D2 surgeries	59	R0 resection rate	R0 resection rate: 86% pCR rate: 20% Median DFS: 32.9 months	(10)
ChiCTR2000030414	II	LAGC	Sindilizumab + XELOX programme(four cycles) + D2 surgeries	30	R0 resection rate	pCR rate: 33.3% R0 resection rate: 100% 1-year OS rate: 93.3%	(11)
ATTRACTION-4	II/III	G/GEJ cancer	Navulizumab+ SOX or CAPOX vs. Placebo + SOX/CAPOX	724	OS、PFS	PFS: 10.45 vs. 8.34 months (HR = 0.68, p=0.0007) OS: 17.45 vs. 17.15 months (p=0.26, not significantly different)	(12)
KEYNOTE-859	III	G/GEJ cancer	Pembrolizumab+(FP or CAPOX programme)vs Placebo + FP or CAPOX	1579	OS (ITT/CPS ≥ 1/CPS ≥ 10)	ITT OS: 12.9 vs. 11.5 months (HR = 0.78, p<0.0001) CPS≥10 OS: 15.7 vs. 11.8 months (HR = 0.65, p<0.0001)	(13)
ORIENT-16	III	G/GEJ cancer	Sindilizumab + CAPOX programme vs Placebo+ CAPOX	650	OS (whole population/CPS ≥5)	Full population OS: 15.2 vs. 12.3 months (HR = 0.766, p=0.0090) CPS ≥5 population OS: 18.4 vs. 12.9 months (HR = 0.660, p=0.0023)	(14)
CheckMate 649	III	G/GEJ cancer	Nivolumab+ XELOX or FOLFOX vs XELOX or FOLFOX	1581	OS、PFS	OS:13.8 vs 11.6 months(HR=0.80, p=0.0002); PFS:7.7vs6.9(HR=0.77)	(15, 16)

EC, Esophageal Cancer; GEJ, Gastroesophageal Junction Cancer; LAGC, Locally Advanced Gastric Cancer; CF, Cisplatin + 5-Fluorouracil; FLOT, Fluorouracil + Leucovorin + Oxaliplatin + Docetaxel; XELOX, Capecitabine + Oxaliplatin; CAPOX, Capecitabine + Oxaliplatin, FP, Fluorouracil + Platin; FOLFOX, leucovorin + fluorouracil + oxaliplatin; OS, Overall Survival; DFS, Disease-Free Survival; PFS, Progression-Free Survival; CPS, Combined Positive Score.

Therefore, identifying reliable predictive biomarkers for treatment efficacy has become a critical issue for improving patient outcomes.

Recent advances in biomarkers and radiomics technology have provided new perspectives for the precision treatment of gastric cancer in the neoadjuvant setting. On one hand, molecular biomarkers in blood or tissue (e.g., SII, miRNA, ASPH) can assist in efficacy assessment by reflecting the host immune status, modulating tumor signaling pathways, or predicting chemotherapy resistance. On the other hand, radiomics combined with deep learning models (e.g., DLDRN) enables the extraction of high-dimensional features from imaging data such as CT and MRI, offering non-invasive tools for predicting tumor downstaging and lymph node metastasis risk, thus facilitating preoperative efficacy evaluation. Moreover, the combined use of targeted therapies (e.g., Claudin 18.2 monoclonal antibodies) and immune checkpoint inhibitors (ICIs) further extends the personalized approach to NACT.

This review aims to summarize the research progress in biomarkers and imaging models for predicting the efficacy of neoadjuvant treatment in GC, providing theoretical support for optimizing treatment strategies and advancing the practice of precision medicine.

## 2 Biomarkers for predicting the efficacy of neoadjuvant treatment in gastric cancer

**Figure 1:**
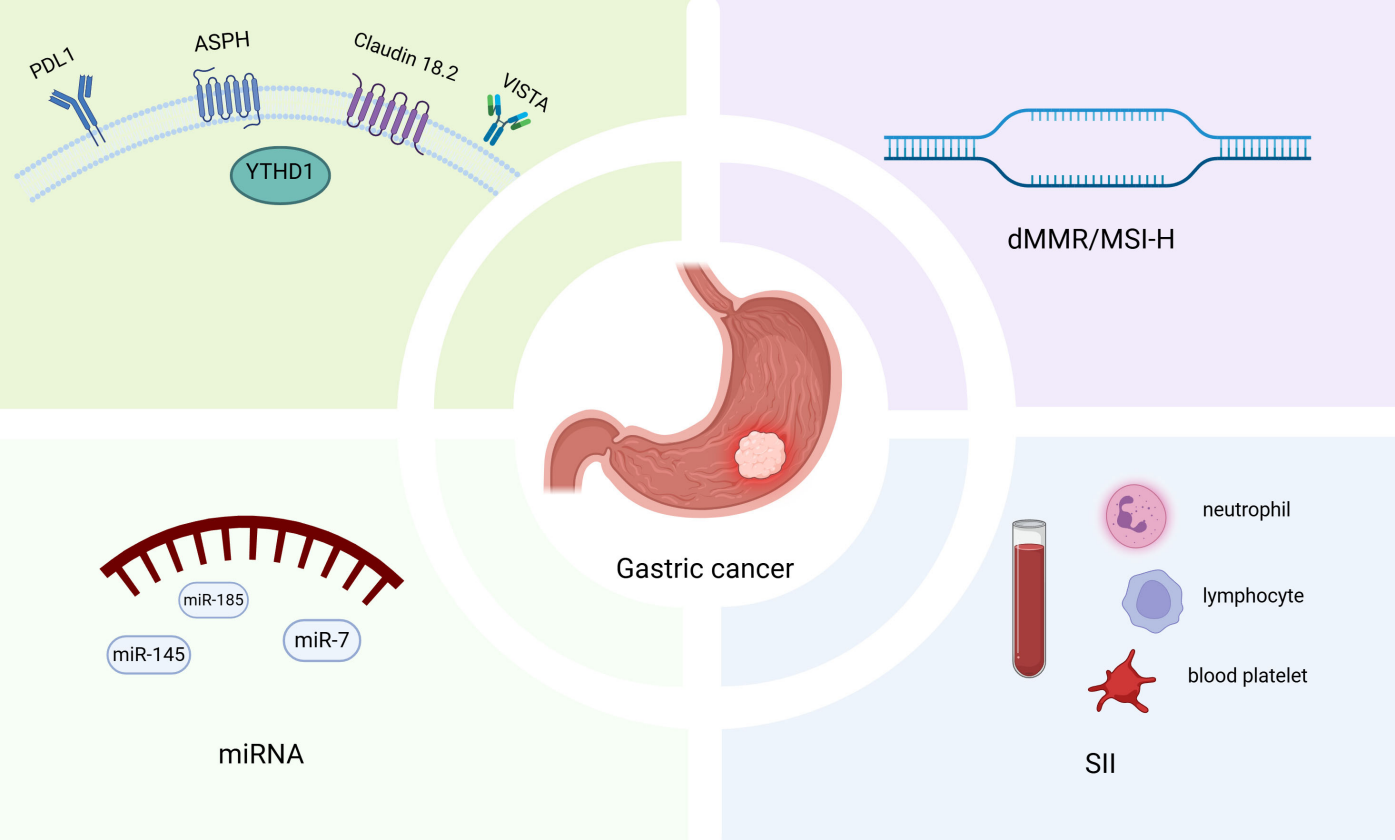
Biomarkers for predicting the efficacy of neoadjuvant treatment in gastric cancer.

In recent years, significant progress has been made in the application of targeted therapy biomarkers in the neoadjuvant treatment of gastric cancer, providing crucial evidence for the development of personalized treatment strategies. Several biomarkers have been validated through clinical trials and integrated into clinical practice. The introduction of molecular markers in blood or tissue, such as systemic immune-inflammation index (SII), PD-L1 expression, and dMMR/MSI-H, has further expanded the patient population eligible for neoadjuvant therapy (**Figure 1**). At the same time, the development of novel therapeutic strategies, including bispecific antibodies and CAR-T cell therapy, holds the potential to extend the scope of targeted therapies, offering more precise neoadjuvant treatment options for patients with LAGC.

### 2.1 Systemic immune-inflammation index

The systemic immune-inflammation index (SII) is a composite inflammatory marker based on the peripheral blood counts of neutrophils, lymphocytes, and platelets, with the following formula: SII = Neutrophil count × Platelet count/Lymphocyte count (17). This index integrates the interactions between innate immunity (neutrophils), adaptive immunity (lymphocytes), and coagulation-inflammation responses (platelets), providing a dynamic reflection of the balance between the host immune microenvironment and systemic inflammation. Numerous clinical studies have shown that systemic inflammatory responses are prevalent in malignancies, and SII, as a quantifiable indicator of inflammatory burden, is closely associated with tumor progression and prognosis. For example, in solid tumors such as lung cancer (18, 19) and colorectal cancer (20, 21), elevated baseline SII levels are significantly correlated with shortened overall survival (OS).

The SII has garnered increasing attention as a prognostic tool in gastrointestinal oncology, with its utility in predicting outcomes following neoadjuvant therapy for GC being actively investigated across diverse geographic populations. Evidence from a Chinese cohort of 140 patients with LAGC demonstrated that a post-treatment decrease in SII (ΔSII < 0) was strongly associated with superior pathological response, showing a significantly higher rate of tumor regression grade (TRG) 0/1 compared to those with ΔSII ≥ 0 (45.2% vs. 19.1%, p = 0.003). This parameter was validated as an independent predictor of favorable pathological response (OR = 6.05, p < 0.001) and was further correlated with significantly improved DFS (p = 0.031) and OS (p = 0.006). Multivariate Cox regression confirmed ΔSII ≥ 0 as an independent adverse prognostic factor for OS (HR = 2.13, 95% CI: 1.22–3.72, p = 0.008) (22). Supporting these findings, Meng et al. (23), established that a baseline SII below 507.45 was associated with prolonged survival in their patient series (P < 0.001).

Notably, the prognostic relevance of SII is not confined to Eastern cohorts. Preliminary investigations from European centers (24) have also begun to report associations between systemic inflammatory markers, including SII-derived ratios, and survival outcomes in Western GC patients, although proposed cutoff values sometimes differ from those reported in Asian populations. This suggests underlying biological consistency while highlighting the influence of ethnic, demographic, or clinicopathological variations.

Collectively, these findings from international cohorts underscore that dynamic monitoring of SII could provide a real-time, non-invasive method for assessing therapeutic efficacy and predicting survival. However, the current evidence body remains constrained by its predominance in retrospective, single-center studies, often with limited sample sizes. A significant barrier to clinical translation is the lack of a standardized, validated threshold for SII change. Future research must prioritize large-scale, international multi-center prospective studies to validate and harmonize SII parameters across diverse populations. Furthermore, exploring the synergistic potential of SII with other inflammatory biomarkers (e.g., CRP, IL-6) could pave the way for developing more robust, globally applicable predictive models. Given its cost-effectiveness and accessibility, SII holds considerable promise for guiding personalized treatment strategies worldwide, pending conclusive validation through concerted international collaboration.

### 2.2 PD-L1

Programmed death ligand 1 (PD-L1), also known as CD274 and B7-H1, is an important immune checkpoint molecule. The role of PD-L1 as an immune checkpoint was first elucidated by Japanese scientist Tasuku Honjo and colleagues, for which they were awarded the Nobel Prize (25, 26). Further studies have shown that cancer cells also express PD-L1, which interacts specifically with the receptor Programmed Death 1 (PD-1) on T cell surfaces. The binding of PD-L1 to PD-1 leads to the inhibition of T cell receptor-mediated lymphocyte proliferation and cytokine secretion (27). Antibodies that block PD-L1 or PD-1 can significantly enhance anti-tumor immunity in both mouse cancer models and human bodies, offering new strategies for tumor immunotherapy (28–31). Building upon this foundation, multiple pivotal clinical trials worldwide—including KEYNOTE-859 in the United States and China’s ORIENT-16 trial—have collectively validated its predictive value in neoadjuvant immunotherapy for GC (13, 14).

In the neoadjuvant treatment of GC,ICIs combined with chemotherapy or radiotherapy have shown superior effects compared to ICIs alone. Patients with high PD-L1 expression tend to benefit more from neoadjuvant therapy with ICIs (31). Furthermore, compared to NACT alone, the combination of PD-1/PD-L1 inhibitors improves pathological complete response (pCR) rates and major pathological response rates. Most adverse events associated with neoadjuvant PD-1/PD-L1 inhibitor therapy are controllable and do not result in treatment delays or mortality (6). The incidence and severity of treatment-related adverse events (TRAEs) associated with ICIs are generally lower than those associated with chemotherapy (32).

### 2.3 MSI-H/dMMR

Deficient mismatch repair (dMMR) and high microsatellite instability (MSI-H) are molecular features found in cancers. Microsatellites are short tandem repeat sequences in the genome, and the mismatch repair system is responsible for correcting errors during DNA replication. When this repair system is defective, the microsatellite sequences undergo length changes, leading to MSI-H (33, 34). MSI-H/dMMR is closely related to the efficacy of ICIs therapy in GC (35, 36). The MSI-H subtype accounts for approximately 15-20% of all gastrointestinal cancers (37). Due to the loss of mismatch repair proteins, these tumors exhibit high levels of tumor mutation burden and a rich infiltration of tumor-infiltrating lymphocytes. In a long-term retrospective study, neoadjuvant therapy with ICIs in GC patients led to higher pCR rates (38), although long-term survival benefits are yet to be fully defined. For patients with LAGC or gastroesophageal junction adenocarcinoma, MSI-H/dMMR is a crucial therapeutic biomarker. Clinical studies have shown that preoperative treatment with nivolumab in combination with low-dose ipilimumab is feasible and safe in resectable locally advanced MSI-H/dMMRGC, with high pCR rates (39).

### 2.4 VISTA

V-domain Ig suppressor of T cell activation (VISTA) is an immune checkpoint receptor expressed on tumor-infiltrating T lymphocytes (TILs) and myeloid cells, leading to the inhibition of T cell activation, proliferation, and cytokine production (40, 41). Despite structural similarities between the extracellular domains of VISTA and PD-L1, these two proteins interfere with different subsets of T lymphocytes. VISTA has two confirmed immunosuppressive binding partners: P-selectin glycoprotein ligand-1 (PSGL-1) and VSIG3 (42, 43). At neutral pH, the interaction between PSGL-1 and VISTA is minimal or absent, whereas in an acidic environment, their binding is more pronounced. This suggests that the PSGL-1/VISTA pathway could be a critical route for inhibiting T cell activation (44), and that antibodies selective for acidic pH could effectively block VISTA-mediated immunosuppression.

VSIG3, a novel VISTA ligand, inhibits T cell proliferation and cytokine and chemokine production by binding to VISTA on activated T cells. The co-inhibitory function of VSIG3 on activated T cells, along with its high expression in colorectal cancer, hepatocellular carcinoma, and intestinal-type GC, suggests that blocking the VSIG3/VISTA pathway represents a promising strategy for cancer immunotherapy (45). Inhibition of VISTA enhances T cell recruitment within the tumor microenvironment and promotes the differentiation of CD8+ T cells into effector T cells. VISTA blockade also increases the multifunctionality of CD8+ T cells, and combined blockade of CTLA-4 and VISTA has shown increased efficacy (46).

The translational potential of targeting the VISTA pathway is being actively explored in early-phase clinical trials, reflecting a growing international interest in this emerging immune checkpoint. Pioneering agents such as the anti-VISTA monoclonal antibody JNJ-61610588 (NCT02671955) and the small molecule antagonist CA-170 (NCT02812875) represent initial efforts to clinically validate VISTA inhibition as a viable therapeutic strategy (47). These trials are crucial for determining the safety profile and initial efficacy of VISTA blockade, both as monotherapy and in potential combination regimens.

Concurrently, a critical dimension of VISTA biology is its role in adaptive therapeutic resistance. Compelling evidence from translational studies indicates that VISTA expression is dynamically upregulated in response to conventional cancer treatments. A key study by Schoop et al. demonstrated that platinum-based NACT significantly induces VISTA expression in GC, with elevated levels observed in both tumor and immune stromal cells post-treatment (48). This phenomenon, corroborated by findings in other malignancies such as ovarian and lung cancers, suggests that VISTA serves as a compensatory immune resistance mechanism. The induction of VISTA following chemotherapy provides a compelling scientific rationale for combining standard cytotoxic agents with VISTA inhibitors. Such a strategy aims to preemptively block this escape pathway, potentially reversing resistance and improving long-term survival outcomes, a hypothesis now being tested in next-generation clinical trials.

### 2.5 Dynamic regulation of miRNAs

MicroRNAs (miRNAs) are a class of endogenous, single-stranded non-coding RNA molecules approximately 18–25 nucleotides in length, which function as key post-transcriptional regulators of gene expression through complementary binding to the 3’-untranslated regions (3’-UTRs) of target mRNAs (49, 50)Over 2,000 miRNAs have been annotated in the miRBase miRNA database (51), these molecules participate in the regulation of diverse biological processes and have emerged as promising liquid biopsy biomarkers for cancer diagnosis, prognostic stratification, and therapeutic response prediction due to their remarkable stability in circulation (52). In the context of gastrointestinal malignancies, miRNA dysregulation has been strongly implicated in modulating chemotherapy sensitivity and resistance (53, 54). Esophageal cancer studies have identified specific miRNA signatures correlated with patient response to neoadjuvant and adjuvant therapies (55). Similarly, in GC, comprehensive profiling has revealed significant alterations in miRNA expression patterns following NAC, supporting their potential role as dynamic indicators of treatment efficacy (56, 57).

Functional validation studies have elucidated the mechanistic roles of specific miRNAs in GC pathophysiology and treatment response. Elevated expression of miR-7 suppresses tumor progression through direct targeting of the Raf-1 oncogene, inhibiting cancer cell proliferation, migration, and angiogenesis while promoting apoptosis - suggesting its potential utility as a biomarker for favorable NACT outcomes.

Functional validation studies have elucidated the mechanistic roles of specific miRNAs in GC pathophysiology and treatment response. Elevated expression of miR-7 suppresses tumor progression through direct targeting of the Raf-1 oncogene, inhibiting cancer cell proliferation, migration, and angiogenesis while promoting apoptosis - suggesting its potential utility as a biomarker for favorable NACT outcomes (58). Additional miRNAs including miR-143-3p, miR-143-5p, and miR-574-3p demonstrate diagnostic value (59), while miR-145 and miR-185 expression patterns show association with both efficacy and adverse event profiles in patients receiving the SOX regimen (S-1 plus oxaliplatin) as neoadjuvant therapy (60). Furthermore, miR-551b-5p has been identified as a promising companion biomarker that may enhance the accuracy of computed tomography (CT)-based evaluation of treatment response (59).

The convergence of artificial intelligence (AI) and liquid biopsy technologies has recently accelerated the development of robust miRNA-based predictive models. Exosomes - nanoscale extracellular vesicles that carry miRNAs and other nucleic acids - provide a non-invasive window into real-time tumor microenvironment dynamics. In an innovative approach, researchers integrated high-throughput exosomal RNA sequencing with AI-driven feature selection algorithms, including LASSO-Cox regression, to identify a six-miRNA signature (containing let-7i-5p, miR-181a-5p, and miR-1307-3p) predictive of NACT response in LAGC. This multi-miRNA model demonstrated consistent performance with an AUC of 0.774 (95% CI: 0.64-0.92) in an independent validation cohort (n=43), highlighting the transformative potential of combining liquid biopsy with machine learning for treatment response prediction (61).

These advances represent a paradigm shift toward integrated multi-modal biomarker platforms. Liquid biopsies, particularly those profiling miRNAs and exosomal RNAs, stand as a cornerstone of this new paradigm, offering a dynamic and non-invasive window into tumor biology. Nevertheless, the translation of these discoveries into routine clinical practice is contingent upon overcoming profound challenges. The lack of standardized detection methodologies across the entire liquid biopsy workflow—from sample collection and processing to RNA quantification and data analysis—introduces significant variability and hinders the establishment of universally applicable diagnostic thresholds. Furthermore, the cost and technical complexity of advanced molecular profiling currently limit the accessibility and scalability of these tests in diverse healthcare settings.

To fully realize the potential of these biomarkers in personalizing neoadjuvant therapy for gastric cancer patients worldwide, a concerted global effort is required. The immediate priorities must include the validation of specific biomarker panels through large-scale, international multi-center prospective trials, which will also address the critical need for benchmarking across diverse populations. Concurrently, the development of robust, cost-effective, and point-of-care detection platforms is imperative to bridge the gap between computational predictions and clinically actionable tools. Only by systematically addressing these analytical and translational hurdles can the promise of liquid biopsies be fully integrated into the future of precision oncology.

### 2.6 Chemotherapy resistance mechanisms mediated by aspartate β-hydroxylase

Aspartate β-hydroxylase (ASPH) has emerged as a compelling therapeutic target in GC due to its central role in tumor progression and treatment resistance. As a highly conserved transmembrane enzyme, ASPH is frequently overexpressed in GC tissues and drives malignant transformation through pleiotropic mechanisms. It potently enhances cancer cell proliferation, migration, and invasive capacity, while simultaneously stimulating tumor angiogenesis and suppressing anti-tumor immunity—effects primarily mediated through constitutive activation of the Notch and SRC signaling pathways (62, 63). The contributory role of ASPH in oncogenesis and metastatic dissemination has been extensively validated across multiple solid tumors, including hepatocellular, pancreatic, and breast carcinomas (64–68).

Recent translational evidence further implicates ASPH in mediating resistance to NACT in gastric cancer. Retrospective analyses have demonstrated that ASPH overexpression correlates significantly with poor pathological response and shortened survival in GC patients receiving platinum-based NACT, suggesting its potential utility not only as a therapeutic target but also as a predictive biomarker for chemoresistance.

Retrospective cohort analysis demonstrated that positive ASPH expression was significantly associated with shorter OS in GC patients receiving NACT (p = 0.0010), with median OS of 18.8 months in ASPH-positive patients versus 55.6 months in ASPH-negative patients. A similar trend was observed in progression-free survival (PFS) (p = 0.0067), with median PFS of 9.7 months and 46.9 months, respectively. Univariate Cox regression analysis identified ASPH expression as a significant prognostic factor (HR = 2.381; 95% CI: 1.275–4.445; p = 0.006), which remained significant in multivariate analysis (HR = 2.804; 95% CI: 1.468–5.356; p = 0.002) (69).

In the broader context of cancer research, these findings align with growing international recognition of ASPH as a significant modulator of tumor behavior. Studies across various cancer types, including hepatocellular carcinoma and pancreatic ductal adenocarcinoma from multiple research institutions worldwide (65), have consistently demonstrated ASPH’s involvement in key oncogenic processes. The conservation of ASPH-related mechanisms across different malignancies and ethnic populations underscores its fundamental role in cancer pathophysiology.

Collectively, these results position ASPH not merely as a predictive biomarker for NACT response assessment, but more importantly as a promising therapeutic target for overcoming chemotherapy resistance. Several international drug development programs are currently exploring ASPH-targeting strategies, with early-phase clinical trials showing potential for enhancing chemosensitivity in gastrointestinal malignancies. This expanding body of global research highlights the translational significance of ASPH in gastric cancer management and its potential applicability across diverse patient populations.

### 2.7 The immunoregulatory potential of YTHDF1

YTHDF1 (YTH N6-methyladenosine RNA binding protein 1), a key cytoplasmic reader of N6-methyladenosine (m6A) modifications, plays a pivotal role in post-transcriptional regulation by facilitating the translation of methylated mRNA transcripts through interactions with translation initiation factors (69). Beyond its fundamental RNA regulatory functions, emerging evidence from international research consortiums has established YTHDF1 as a critical modulator of tumor immunology and cancer progression (70–72). Comprehensive multi-omics analyses across different cancer types have demonstrated that YTHDF1 significantly influences the tumor immune microenvironment by regulating the infiltration and functional states of cytotoxic immune cells. Studies from research groups have consistently shown that YTHDF1 impairs the anti-tumor activity of CD8+ T cells and natural killer (NK) cells, thereby fostering an immunosuppressive niche. This immunosuppressive function, coupled with YTHDF1’s involvement in multiple oncogenic signaling pathways, positions it as a promising therapeutic target (73–76). Blocking YTHDF1 can re-activate suppressed anti-tumor immunity and synergistically enhance the therapeutic effects of anti-PD-L1 inhibitors (77–79).

In GC, mechanistic studies have elucidated a hypoxia-mediated regulatory axis where HIF-1α binds to the H19 promoter, inducing H19 overexpression that subsequently activates YTHDF1/SCARB1 signaling. This pathway promotes GC cell proliferation, migration, and angiogenesis, identifying YTHDF1 as a promising target for anti-angiogenesis therapies in gastric malignancies. The conservation of YTHDF1-related mechanisms across diverse cancer types and ethnic populations underscores its fundamental importance in cancer biology and highlights its potential as a broad-spectrum therapeutic target in oncology.YTHDF1 modulates GC through multiple signaling pathways (80–84), and targeting YTHDF1 is considered a potential therapeutic strategy. In GC patients, high expression of YTHDF1 is associated with poorer survival outcomes (80). YTHDF1 promotes tumorigenesis and metastasis in GC by enhancing the translation of USP14 protein in an m6A-dependent manner, making it a potential target for GC treatment (85). Overexpression of YTHDF1 in GC promotes tumor growth by inducing cell proliferation and inhibiting dendritic cell-mediated anti-tumor immune responses. During neoadjuvant therapy, modulation of YTHDF1 expression or activity may influence the proliferation, invasion, and metastasis of GC cells, thereby enhancing the efficacy of neoadjuvant therapy.

The convergence of evidence from multiple research centers worldwide solidifies YTHDF1’s position as both a key pathogenic driver and a compelling therapeutic target in gastric cancer. Ongoing preclinical investigations across different geographical regions are currently exploring various YTHDF1-targeting strategies, with particular focus on their potential to enhance treatment responses in neoadjuvant settings and overcome therapeutic resistance mechanisms.

### 2.8 Clinical translation of Claudin 18.2

Claudin 18.2 is a tight junction protein and a subtype of the Claudin protein family. Under normal conditions, it is expressed at low levels in the differentiated epithelial cells of the gastric mucosa, playing a crucial role in maintaining intercellular junctions. It is also involved in maintaining the barrier function of the gastric mucosa, preventing the leakage of H+ ions (86). In the oncological context, Claudin 18.2 undergoes notable dysregulation, with its overexpression being frequently observed in gastric cancer, especially in gastric adenocarcinoma. This expression pattern has been consistently validated across multiple international patient cohorts and is recognized as a hallmark molecular alteration in gastric carcinogenesis (87, 88). Mechanistic studies from research institutions worldwide have revealed that Claudin 18.2 engages with multiple oncogenic signaling pathways, potentially facilitating tumor progression and malignant transformation (89–92). During malignant transformation, Claudin 18.2 becomes specifically overexpressed on the surface of tumor cells, making it an ideal target for precision therapy. Significant breakthroughs in this field have been achieved through international multicenter clinical investigations.

Zolbetuximab, as the first monoclonal antibody targeting Claudin 18.2, has been validated in two phase III clinical trials. Study data demonstrate that this drug combined with standard chemotherapy regimens significantly improves both OS and PFS in patients with Claudin 18.2-positive advanced gastric cancer (89, 93, 94). The subsequent SPOTLIGHT phase III trial further confirmed that in patients with Claudin 18.2-positive, HER2-negative advanced gastric cancer, Zolbetuximab combined with first-line chemotherapy improved both PFS and OS, providing a new therapeutic option for this patient population (95).

Concurrently, other Claudin 18.2-targeted therapies have shown promising results. Claudiximab, a novel chimeric IgG1 monoclonal antibody, demonstrated in the phase IIb FAST clinical trial that its combination with chemotherapy significantly improved OS and PFS in Claudin 18.2-positive GC patients (96). These research achievements mark the formal entry of Claudin 18.2-targeted therapy into clinical practice. Building upon these positive outcomes, global research institutions are actively exploring more advanced targeting strategies. Currently, various bispecific antibodies, antibody-drug conjugates, and Claudin 18.2-targeted CAR-T cell therapies have entered clinical evaluation stages. These innovative therapeutic approaches are expected to further expand treatment options for Claudin 18.2-positive GC patients.

This series of clinically advancements, driven by international multicenter collaborations, not only establishes Claudin 18.2 as an important therapeutic target in gastric cancer but also demonstrates the substantial potential of molecular subtype-guided precision therapy in improving GC outcomes.

### 2.9 Current clinical guideline status and translational trajectory of biomarkers

The biomarkers for predicting efficacy of neoadjuvant therapy in GC can be broadly categorized into two groups based on their level of clinical validation and guideline endorsement: guideline-endorsed standard biomarkers and investigational biomarkers with translational potential (**Table 2**).

**Table 2:** Biomarkers related to the prediction of efficacy of neoadjuvant therapy in gastric cancer.

Research	Mechanism of action	Application	Limitations and perspectives	Reference
Systemic Immunoinflammatory Index (SII)	Integration of immune and coagulation inflammatory responses reflecting the immune microenvironment and inflammatory state	ΔSII<0 Patients with high tumor remission and long survival predict efficacy and survival	Small sample size, unstandardized thresholds, multi-center validation and exploration of joint applications required	(17–24)
PD-L1 and dMMR/MSI-H	PD-L1 inhibits T cells; dMMR/MSI-H gives tumors high mutational load and abundant lymphocytes	Patients with high PD-L1 expression or dMMR/MSI-H have high remission rates with ICIs.	Long-term survival benefit unclear	(25–32)
VISTA	Expressed in T lymphocytes and myeloid cells, inhibits T cell function	Combined blockade of VISTA and PD-1 promotes tumor regression and its expression is associated with drug resistance after neoadjuvant chemotherapy	Mechanism of action and *in vivo* relevance of PSGL-1 to be investigated	(40–48)
MicroRNAs (miRNAs)	Regulates gene expression by binding mRNA	Multiple miRNAs can assess efficacy, predict chemotherapy outcomes, exosomal RNA and AI-based models have potential	Mostly in exploratory phase, some clinical value to be verified	(49–61)
Aspartate β-hydroxylase (ASH)	Activation of Notch and SRC pathways to promote tumor growth and drug resistance	Positive expression is associated with short patient survival and may mediate chemotherapy resistance	Feasibility and efficacy as a therapeutic target to be studied	(62–68)
Immunomodulatory potential of YTHDF1	Recognition of RNA methylation markers, affecting immune cell function	High expression is associated with poor survival in gastric cancer patients, or may modulate treatment efficacy	More research needed to validate in neoadjuvant therapy for gastric cancer	(69–85)
Clinical translation of Claudin 18.2	Highly expressed in gastric cancer and involved in tumor progression	Drug targeting Claudin 18.2 combined with chemotherapy improves patient survival	Some treatment strategies are in clinical trials	(86–96)

PD-L1 and MSI-H/dMMR: These two biomarker classes have been formally recommended by the Chinese Society of Clinical Oncology (CSCO) guidelines, the National Comprehensive Cancer Network (NCCN) guidelines, and the European Society for Medical Oncology (ESMO) guidelines for informing immunotherapy decisions in advanced gastric cancer. Building upon their established predictive value in the metastatic setting, their application in the neoadjuvant immuno-therapeutic context is under intensive investigation. Their incorporation as stratification or enrollment factors in major clinical trials (e.g., KEYNOTE-585, CHECKMATE-649) underscores their status as the current cornerstone of the clinical predictive framework closest to routine application.

The majority of biomarkers discussed in this review, such as the SII, miRNAs, ASPH, VISTA, and YTHDF1, currently fall within this category. Although promising predictive potential has been demonstrated in retrospective studies, none are currently recommended by major international guidelines for clinical decision-making. The primary barriers to their clinical adoption include a lack of validation in prospective, multi-center trials and the absence of standardized detection methodologies. For instance, optimal cut-off values for SII and specific, validated miRNA signatures require further elucidation. Claudin 18.2 represents a special case. It is itself a CSCO and internationally recognized therapeutic target. The success of phase III trials (SPOTLIGHT, GLOW) investigating Zolbetuximab has firmly established its role in advanced disease. Its potential value in the perioperative setting is now being actively explored, positioning it to potentially evolve from a “therapeutic target” into a complementary “predictive biomarker.”

In summary, the prediction of efficacy in GC neoadjuvant therapy is evolving from traditional histopathological assessment towards a multidimensional precision oncology paradigm. PD-L1 and MSI-H/dMMR serve as the current foundational elements of clinical guidelines, providing an initial framework for patient stratification. However, tumor heterogeneity and primary/acquired resistance necessitate the discovery of additional biomarkers. The investigational biomarkers detailed in this review, such as SII, miRNAs, and ASPH, although not yet included in guidelines, offer complementary insights from diverse biological angles—including systemic inflammation, epigenetic regulation, and metabolic drug resistance—thereby revealing substantial potential for clinical application. The paramount task for the future is the rigorous validation of these promising candidates within prospective clinical trials, ultimately facilitating their integration into clinical practice guidelines and enabling truly individualized neoadjuvant treatment strategies.

## 3 The role of imaging in predicting the efficacy of neoadjuvant therapy in gastric cancer

Radiomics has emerged as a globally recognized interdisciplinary field that leverages advanced computational image processing and machine learning to extract high-throughput quantitative features from standard medical images including CT, MRI, and PET-CT. These sub-visual features encode rich biological and pathological information about tumors that cannot be discerned by human observation alone. Through sophisticated algorithms, radiomics uncovers intrinsic patterns correlating with disease pathogenesis, progression dynamics, and ultimate therapeutic response, providing a powerful non-invasive approach for treatment assessment.

In the specific context of predicting neoadjuvant therapy efficacy for GC, international research consortia have demonstrated that radiomic analysis of serial imaging data—acquired before and during treatment—can identify robust signatures predictive of treatment response, pathological tumor regression, and long-term patient outcomes. This paradigm, validated across diverse patient populations in Europe, North America, and Asia, offers clinicians worldwide an evidence-based framework to guide personalized therapeutic strategies. However, the field continues to address key challenges in feature standardization and cross-scanner reproducibility through initiatives such as the Image Biomarker Standardization Initiative (IBSI) (97), underscoring the importance of global collaboration in translating these advanced analytical tools into routine clinical practice across different healthcare systems.

### 3.1 Technical foundations of radiomics

#### 3.1.1 Data preprocessing

Raw medical imaging data often contain noise and artifacts, and images obtained from different devices or with varying scanning parameters may differ in grayscale values and resolution, requiring preprocessing (98). Common image denoising techniques include Gaussian filtering and median filtering, which remove noise and enhance the signal-to-noise ratio. Image normalization, achieved through linear or nonlinear transformations, standardizes grayscale values and eliminates discrepancies caused by device differences. Image registration is used for multimodal imaging, aligning images from different modalities in space to facilitate subsequent analysis. Image segmentation is a crucial step. Traditional methods such as manual segmentation, thresholding, and region-growing methods are inefficient and subjective. Nowadays, cutting-edge technologies such as deep learning-based convolutional neural networks (CNNs) can achieve fast, high-precision segmentation, remove image noise (99–101), and enhance research efficiency and accuracy.

Notwithstanding the promising predictive accuracy demonstrated in numerous studies, the widespread clinical adoption of radiomics faces substantial practical hurdles that must be squarely addressed. The reproducibility of radiomic features is critically dependent on standardized imaging acquisition protocols across different scanner manufacturers and institutions, a goal that remains challenging to achieve in routine practice. Initiatives like the IBSI are pivotal for establishing methodological consistency (97). Furthermore, the transition from a research model to a clinical tool necessitates robust external validation in multi-center cohorts to overcome center-specific bias and prove generalizability. Finally, seamless integration into clinical workflows requires solving challenges related to automated segmentation, computational efficiency, model interpretability for clinicians, and ultimately, demonstrating improved patient outcomes in prospective trials to justify its implementation. Acknowledging these barriers is essential for guiding the field towards clinically viable and impactful solutions.

### 3.2 Construction of prognostic models based on imagingomics

In the context of radiomics for predicting neoadjuvant treatment efficacy in GC, extensive raw features extracted from medical images often contain redundant or irrelevant information. Feature analysis and selection are therefore essential, typically employing methods such as correlation analysis (to filter features with low correlation to outcomes like pathological response or PFS and principal component analysis (PCA) to reduce dimensionality while retaining key information (102). Using the selected features, machine learning models—including support vector machines (SVM) (103), random forest (RF) (104), and logistic regression (LR) (105)—are constructed. Model parameters are optimized via techniques like grid search or random search to enhance performance. Validation involves independent test datasets and evaluation metrics (accuracy, sensitivity, specificity, AUC) to assess predictive capability and reliability, with cross-validation employed to mitigate overfitting (102–107).

### 3.3 Research progress in imagingomics based on enhanced CT for predicting neoadjuvant treatment efficacy in gastric cancer

CT (Computed Tomography) is a crucial imaging diagnostic tool in medicine, based on X-ray and computer technology. During the examination, the X-ray beams emitted by the X-ray tube pass through the body area under investigation after being calibrated by the collimator. Due to differences in tissue density, the X-rays are attenuated to varying degrees and received by the detectors, which convert them into electrical and digital signals. In the scanning process, the X-ray tube and detectors rotate around the patient’s body, acquiring attenuation data from multiple angles. Subsequently, algorithms such as filtered back projection are used by computers to process the data and reconstruct cross-sectional images of the body, displayed as grayscale on a monitor (108, 109). With this principle, CT provides precise anatomical information for disease diagnosis.

Contrast-Enhanced CT (CECT) is the preferred imaging method for diagnosing the TNM staging of gastric cancer before neoadjuvant chemotherapy and can also be used to evaluate the downstaging of tumors after neoadjuvant chemotherapy (110). Dual-Energy CT (DECT) can quantify the differing densities of mixed substances by acquiring two different energy levels, providing better assessment of tumor invasion depth and patient response to neoadjuvant chemotherapy. MRI, which involves no radiation, offers rich and detailed imaging information for tumor staging in gastric cancer through various imaging sequences. Therefore, different imaging techniques may influence the accuracy of machine learning in the initial diagnosis of gastric cancer or in the imaging of gastric lesions after neoadjuvant chemotherapy.

In recent years, radiomics analysis based on enhanced CT has played an important role in the treatment of various cancers (111–113) and has shown significant potential in predicting the efficacy of NACT in LAGC. Studies by Cui et al. (114–117) have further provided new insights and methods for applying CT in predicting the efficacy of neoadjuvant treatment in GC through the construction of a deep learning radiomics nomogram (DLRN).

Further research has shown that radiomics models can extract high-dimensional features from tumors and lymph nodes, dynamically reflecting chemotherapy-induced microenvironment changes, which provide key information for personalized treatment. Previous studies have primarily focused on the primary tumor site, while the role of lymph node radiomics in efficacy prediction has not yet been clarified. Research by Han et al. combined three radiomics features to construct models for tumor radiomics, lymph node radiomics, and a combined model. They further built a nomogram model incorporating clinical factors. In identifying good responders and poor responders, the tumor radiomics feature model outperformed the lymph node radiomics feature model (AUC in the training cohort: 0.700 vs. 0.637; validation cohort: 0.688 vs. 0.648). However, the tumor-lymph node radiomics feature model, which combined both tumor and lymph node radiomics, had a higher predictive value, with AUCs of 0.755 and 0.744 in the training and validation cohorts, respectively (118).

Zhong et al. (119) constructed a deep learning delta radiomics nomogram (DLDRN) through a prospective study, focusing for the first time on predicting the response of metastatic lymph nodes. The model achieved an AUC of 0.94 in the validation cohort (sensitivity 78%, specificity 97%), significantly outperforming conventional CT assessment criteria. In the same patient cohort, the commonly used metric of percentage change in the short axis of the largest lymph node yielded a markedly lower predictive value (AUC = 0.37), underscoring the limitation of relying on size-based morphological changes alone and highlighting the superior capability of deep learning radiomics in capturing underlying pathological response. In the prediction of drug resistance, deep learning models based on CT can effectively identify patients with NACT resistance, thus avoiding ineffective treatments (120). These models can identify patients with LAGC who are resistant to NACT, providing valuable information for personalized treatment. Additionally, radiomics has shown remarkable potential in early detection of pathological downstaging (pDS) and in evaluating early chemotherapy efficacy in patients with peritoneal metastasis. For example, a linear discriminant analysis (LDA) model based on 18 features significantly optimized clinical decision-making (121).

### 3.4 Role of MRI imaging modality in the prediction of neoadjuvant treatment efficacy in gastric cancer

Magnetic resonance imaging (MRI) generates images based on the properties of atomic nuclei in a strong magnetic field. The hydrogen nuclei in the human body have magnetic properties and generate a specific precessional frequency in a strong magnetic field. By applying radiofrequency pulses at specific frequencies, hydrogen nuclei absorb energy and undergo resonance. When the radiofrequency pulse stops, the hydrogen nuclei release energy, producing magnetic resonance signals. These signals are received and processed by a computer to form images reflecting the internal structures of the body (122–124).

GC exhibits a high contrast of soft tissues, and MRI can provide imaging with multi-angle, multi-directional, and multi-parameter capabilities. With the development of fast sequences, MRI offers high-resolution images and key information for lesion localization and qualitative diagnosis without radiation hazards (125). MRI is used in the staging of GC, focusing on detecting serosal invasion and lymph node metastasis (126). It has the advantage of being non-invasive; however, issues such as gastrointestinal motility, respiratory motion artifacts, and changes in gastric wall thickness depending on the state of gastric distension can affect imaging. These challenges can be addressed through methods such as drinking water to distend the stomach, using medications to reduce motility artifacts, breath-holding techniques, and multi-planar imaging (127, 128).

MRI, with its excellent soft tissue contrast and multi-parametric functional imaging capabilities, provides unique biological and morphological information for predicting the efficacy of NACT in GC. Compared to DECT, Multiparametric MRI (mpMRI) has higher accuracy in T staging (61%-77%) regardless of tumor invasion depth, outperforming DECT (50%-64%, P < 0.05) (111, 112). Its functional imaging techniques, such as diffusion-weighted imaging (DWI) and dynamic contrast-enhanced MRI (DCE-MRI), can capture treatment responses earlier than morphological changes, driving innovations in personalized treatment decisions (129).

MRI integrates the synergistic advantages of both anatomical and functional imaging. On the one hand, high-resolution T2-weighted imaging (T2WI) allows for clear differentiation of the gastric wall layers, including the mucosal, muscular, and serosal layers, enabling accurate assessment of tumor infiltration depth (T stage). On the other hand, functional imaging techniques can capture early treatment responses before morphological changes are detectable.

Diffusion-weighted imaging (DWI) reflects tumor cell density and microstructure by detecting the diffusion of water molecules. Preliminary studies by FU et al. have shown that deep learning-based radiomic features extracted from pre-treatment DWI images, using transfer learning, exhibit significantly superior predictive performance in assessing the treatment response, pathological response, and lymph node metastasis of locally advanced rectal cancer (LARC) patients following neoadjuvant chemoradiotherapy, compared to features manually designed for radiomics (130–135). The apparent diffusion coefficient (ADC) is a core quantitative parameter in DWI, with low ADC values correlating with high cellular density and proliferative activity. A prospective study (n=32) found that the change in ADC (ΔADC) before and after neoadjuvant chemotherapy was negatively correlated with tumor regression grade (TRG) (r = −0.71, P = 0.000004) (136). Furthermore, ADC values were negatively correlated with Ki-67 index (r=−0.725, p<0.05), suggesting that ADC could indirectly reflect tumor proliferative activity (137).

DCE-MRI quantifies tumor microvascular permeability and blood flow by assessing the dynamic contrast agent uptake, thereby evaluating the sensitivity to anti-angiogenic therapies (138, 139). In a study by Zhu et al. (n=129), DCE-MRI demonstrated effective diagnostic value in evaluating treatment responses to NACT in LAGC patients (AUC = 0.875, sensitivity 71%, specificity 91.7%, P<0.001) (140).

In radiomics-driven efficacy prediction models, mp-MRI, which integrates DWI, DCE-MRI, and T2WI features, provides a comprehensive analysis of tumor heterogeneity. The advanced mp-MRI strategy can serve as a valuable tool for restaging GC after neoadjuvant therapy, enabling accurate differentiation of patients with favorable responses to neoadjuvant therapy, which has significant clinical implications for personalized treatment strategies and improving patient prognosis (141).

Tumor Regression Grade (TRG) is a pathological or radiological grading system used to assess the extent of reduction in tumor cell number or structural disruption within the primary lesion following neoadjuvant treatment (such as chemotherapy, radiotherapy, or chemoradiotherapy). TRG has significant prognostic value. However, the TRG assessment is typically only available post-surgery, which limits its clinical application for predictive purposes. Several studies have shown that MRI-based tumor regression grading (mrTRG) shows promise in evaluating the effectiveness of neoadjuvant therapy, including parameters such as pCR and neoadjuvant response scoring (NAR).

MRI radiomics has proven beneficial not only in GC patients but also in assessing treatment prognosis for locally advanced non-small cell lung cancer (NSCLC) (142)and locally advanced cervical cancer (143).

While the reported AUCs of radiomics models are promising, their incremental value must be judged against the current clinical benchmark—histopathological evaluation of the resection specimen (TRG and pCR). It is critical to note that radiomics does not seek to replace this gold standard but to provide a preoperative, non-invasive surrogate. The clinical utility lies in its potential to identify non-responders early, thereby avoiding ineffective therapy and unnecessary surgical delays, a decision that cannot be made by postoperative pathology.

In summary, while radiomics demonstrates clear superior predictive accuracy over conventional size-based imaging criteria, its translation into routine practice hinges on overcoming challenges of standardization and validation. Its ultimate clinical role is not as a standalone test, but as a powerful complement to existing pathological and clinical frameworks, aiming to enable earlier and more personalized therapeutic decision-making (**Table 3**).

**Table 3:** Imaging models in neoadjuvant therapy for gastric cancer.

Classifications	Research	Mechanism of action	Application	Limitations and perspectives	Reference
Radiomics	Construction of prognostic models based on CT imaging histology (e.g. DLDRN, DLCS)	Data preprocessing, feature extraction and analysis, model construction and validation	Models such as DLDRN predict tumor response, survival risk and pathological downstaging	Single center, sample size limitation, unclear correlation between imaging and biology, multi-center validation and synergistic application to be studied	(108–121)
Radiomics	The role of MRI imaging modalities in efficacy prediction	Imaging using the properties of atomic nuclei with multiple advantages and functional imaging techniques	Techniques such as DWI and DCE-MRI can assess tumors and mrTRG is effective in assessing neoadjuvant therapy	Equipment affects feature reproducibility, small sample sizes, inability to survive analyses, and limitations of single-level analyses	(122–143)
Synergy between biomarkers and imaging histology	Joint model construction and application	Biomarkers Reveal Intrinsic Mechanisms, Imaging Histology Resolves Spatial Heterogeneity	Combined approach predicts pCR, assesses tumor marker expression status	Need to explore more effective joint approaches to improve forecast accuracy and usefulness	(144–151)

## 4 Synergy between biomarkers and imaging histology

**Figure 2:**
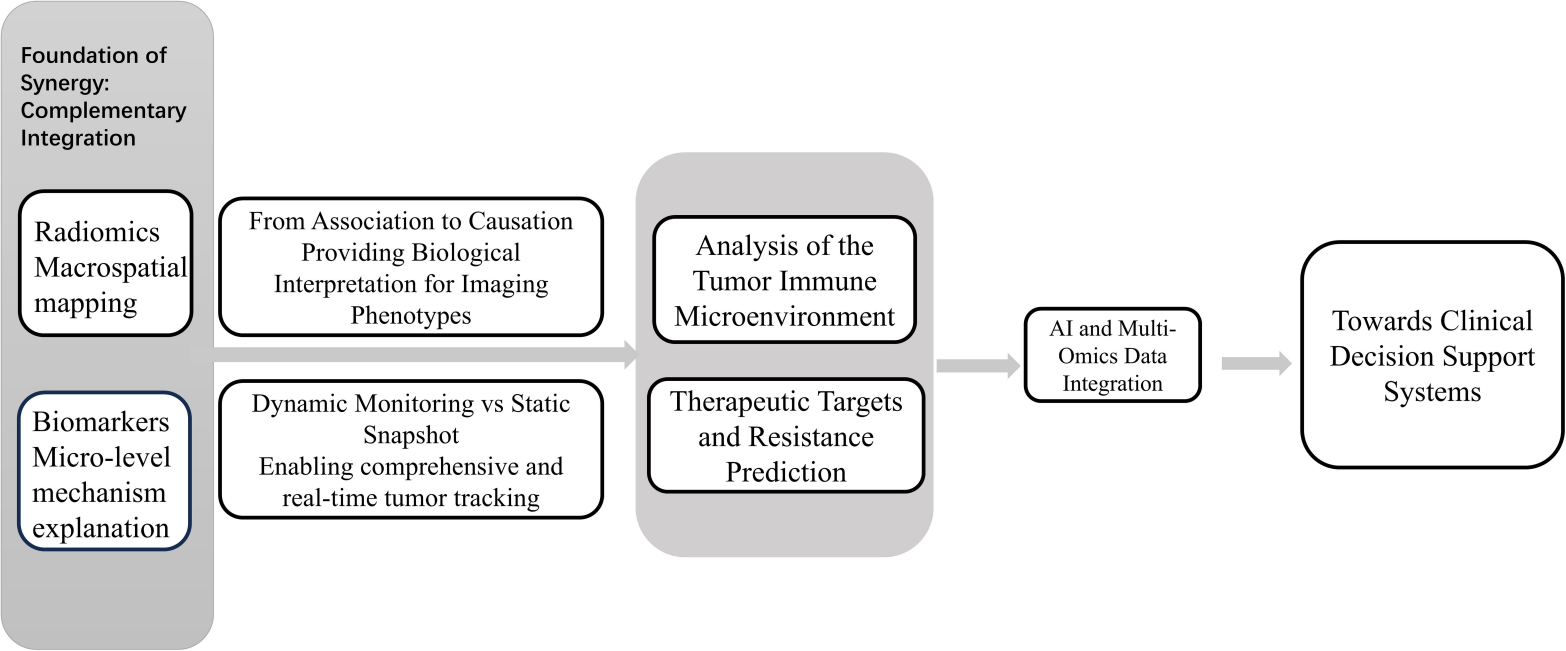
Synergy between biomarkers and imaging histology.

In the evolving landscape of precision oncology, the integration of radiomics and biomarkers represents a transformative shift from traditional, unidimensional diagnostic approaches towards a holistic, systems-level understanding of cancer biology. This synergy transcends mere technical complementarity, establishing a new paradigm where macroscopic imaging phenotypes and molecular signatures are dynamically correlated to decode tumor heterogeneity, track disease progression, and predict therapeutic response with unprecedented spatial and temporal resolution (**Figure 2**).

Radiomics functions as a non-invasive spatial cartographer of the entire tumor landscape. By extracting vast arrays of quantitative features from standard medical images (CT, MRI, PET), it encodes information about tumor texture, shape, and intensity distribution that is imperceptible to the human eye (144). This macroscopic profiling captures the intricate heterogeneity within and around the tumor mass, effectively overcoming the sampling bias inherent in tissue biopsies (145, 146). Conversely, molecular biomarkers—encompassing genomic, transcriptomic, proteomic, and metabolomic analytes—serve as mechanistic interpreters, revealing the fundamental drivers of oncogenesis, proliferation, immune evasion, and drug resistance. They provide the “why” behind the “what” that imaging depicts.

The true power of this alliance lies in their bidirectional interaction (147, 148). Radiomic features are increasingly recognized as non-invasive surrogates for specific molecular and cellular processes. For instance, certain texture patterns on CT have been robustly linked to the expression of immune checkpoints like PD-L1, the mutational status of key oncogenes, or the density of tumor-infiltrating lymphocytes (e.g., CD8+ T cells) (149–151). This allows for the inference of a tumor’s “immune contexture” and its potential responsiveness to immunotherapy without repeated invasive procedures. Furthermore, longitudinal radiomic analysis (delta-radiomics) can dynamically monitor shifts in the tumor microenvironment (TME) in response to therapy, capturing early signs of resistance or efficacy that precede morphological changes.

Simultaneously, biomarker data provide the essential biological context to validate and interpret radiomic signatures. The identification of specific genetic alterations or pathway activations helps explain why certain imaging phenotypes are associated with particular clinical outcomes, moving the field from empirical correlation toward causal understanding. This is crucial for developing “radiogenomic” maps, where specific imaging traits are anchored to underlying genomic drivers.

The operationalization of this synergy is particularly evident in two pivotal applications: the non-invasive profiling of the tumor immune microenvironment, and the prediction of resistance to conventional therapies. These applications demonstrate how radiomics and biomarkers converge to create a more comprehensive diagnostic and prognostic framework.

### 4.1 Non-invasive profiling of the tumor immune microenvironment

The cellular composition and functional state of the tumor immune microenvironment are critical determinants of immunotherapy response. While biomarkers such as PD-L1 immunohistochemistry, tumor mutational burden (TMB), and MSI status provide crucial molecular insights, they are often limited by tumor heterogeneity and the static nature of a single biopsy. Radiomics addresses these limitations by capturing the spatial architecture and heterogeneity of the entire tumor immune landscape (147, 149, 152, 153).

The complex spatial and functional heterogeneity of the TME in GC presents a major challenge for treatment stratification and response prediction. Deep learning radiomics has emerged as a powerful, non-invasive methodology to address this challenge by decoding TME characteristics directly from standard medical images. This approach enables comprehensive profiling of the entire tumor landscape, overcoming the sampling biases inherent in single-site biopsies and providing dynamic insights into TME evolution during therapy. Within this rapidly advancing field, several studies have demonstrated the capacity of radiomic signatures to serve as surrogate biomarkers for specific TME components and functional states.

Exemplifying this approach, the study by Sun et al. systematically integrated multi-scale patient data—including quantitative imaging features, transcriptomic profiles, and tumor immune phenotypes—to develop an eight-variable radiomic signature predictive of CD8+ T-cell infiltration. This signature was rigorously validated against CD8A gene expression data from The Cancer Genome Atlas (TCGA) cohort, achieving statistically significant predictive accuracy (AUC = 0.67, p = 0.0019). Furthermore, in a cohort with predefined immune phenotypes, the radiomic model effectively discriminated between immune-inflamed and immune-desert tumors (AUC = 0.76, 95% CI: 0.66–0.86, p < 0.0001) (154), demonstrating the potential of non-invasive imaging to resolve key elements of the tumor immune microenvironment. The combination of intratumoral and peritumoral radiomics methods has been shown to effectively predict pCR (155), with distinct radiomic response features observed for different molecular subtypes. Han et al. used a machine learning-based radiomic approach to assess TME phenotypes and predict responses to anti-PD-1/PD-L1 immunotherapy. The constructed radiomic features demonstrated good performance across multiple cohorts, effectively distinguishing TME phenotypes and predicting immunotherapy responses (147). Additionally, the study reported that combinations of intratumoral and peritumoral features were associated with TILs, and an imaging biomarker for the neutrophil-to-lymphocyte ratio (NLR) in the tumor immune microenvironment was developed using intratumoral and peritumoral features from CT images. This biomarker was further studied for its potential predictive ability for prognosis and response to anti-PD-1 immunotherapy (156).

### 4.2 Predicting therapeutic targets and resistance mechanisms

Beyond the immune context, the synergy between radiomics and biomarkers is proving invaluable across the therapeutic spectrum—from predicting the expression of actionable therapeutic targets to deciphering mechanisms of resistance to conventional therapies. The identification of key biomarkers, such as the overexpression of growth factor receptors (e.g., HER2), mutations in driver genes, or upregulation of DNA damage repair (DDR) pathways, traditionally relies on invasive tissue sampling, which can be constrained by spatial heterogeneity and temporal evolution. Radiomics offers a complementary, non-invasive approach to infer the spatial distribution and functional status of these critical pathways across the entire tumor landscape, providing a holistic view that can guide both targeted and conventional chemotherapy.

Furthermore, radiomic features can also reflect cancer gene expression to some extent (157). Extending this paradigm beyond immune contexts, radiomic features have also shown the capacity to infer the expression of specific cancer-related genes involved in therapeutic resistance. For instance, Wu and colleagues constructed a CT-based radiomic model to predict the expression levels of RAD51D and XRCC2, key players in DNA damage repair pathways. The model maintained its predictive performance in an external validation cohort, suggesting that radiomics can serve as a non-invasive proxy for assessing the expression of these genes. This approach holds particular clinical relevance in GC, as elevated expression of RAD51D and XRCC2 may indicate enhanced platinum-based DNA damage repair, thereby identifying patients with inherent resistance to platinum chemotherapy and guiding alternative treatment selection.

Current histological methods used to assess the immune environment and treatment outcomes require tissue acquisition, which may be insufficient for patients receiving neoadjuvant therapy or those with metastatic disease. Additionally, when performed on small biopsies, this approach is subject to sampling bias due to intratumoral spatial heterogeneity. Deep learning in radiomics can provide additional assistance for histological diagnosis without increasing costs or causing harm, thereby effectively enhancing clinicians’ decision-making capabilities. On the other hand, biomarkers that are easily accessible have the advantage of serving as potential alternatives to histological examinations when tissue access is limited or unattainable. Combining these two approaches for individualized immunotherapy decisions in patients can facilitate clinical prognosis judgment.

Radiomics can also be integrated with metabolic and proliferative markers. A study by Chen et al. developed a PET/CT radiomic model based on visceral adipose tissue (VAT) to assess its predictive value for Her-2 (AUC = 0.84) and Ki-67 (AUC = 0.86) expression status in gastric cancer patients. The results showed a significant correlation between the metabolic characteristics of VAT and the expression of Ki-67 and Her-2, supporting the feasibility of using VAT radiomic features to provide non-invasive personalized predictive information for GC patients, complementing conventional clinical decision-making methods (158).

In summary, this chapter has systematically elucidated the transformative potential arising from the integration of radiomics and molecular biomarkers, moving beyond their independent applications to establish a new paradigm in systems oncology. As conceptualized in **Figure 2**, the synergy is fundamentally rooted in a complementary fusion, where the macroscopic, spatial profiling capabilities of radiomics seamlessly intersect with the microscopic, mechanistic insights provided by biomarkers. This is not a mere parallel application but a deep integration that creates a virtuous cycle: radiomic features identify spatially heterogeneous phenotypes of clinical interest, while biomarker data anchor these phenotypes to their underlying biological drivers, thereby validating the imaging signatures and enabling their biological interpretation.

The operationalization of this framework was demonstrated through its pivotal applications in two critical domains. First, in the non-invasive profiling of the tumor immune microenvironment, studies such as that by Sun et al. have established that radiomic signatures can serve as accurate surrogates for specific immune cell infiltrates, effectively discriminating between immunologically “hot” and “cold” tumors. This provides a spatial context that complements single-site biomarker assays like PD-L1 immunohistochemistry. Second, in predicting therapeutic targets and resistance mechanisms, the synergy proves equally powerful. Research encompassing the prediction of HER2 expression and DNA damage repair gene activity (e.g., RAD51D/XRCC2) illustrates how radiomics can non-invasively infer the functional status of key pathways, guiding both targeted therapy selection and identifying innate resistance to conventional chemotherapy.

The convergence of these fields, accelerated by artificial intelligence for multi-modal data integration, is poised to redefine clinical decision-making. It enables a shift from reactive, static assessments towards a proactive, dynamic, and spatially informed approach to cancer management. The resulting composite models enhance the accuracy of patient stratification, early response evaluation, and prognosis, thereby solidifying the role of the radiomics-biomarker synergy as a cornerstone of next-generation precision oncology.

## 5 Conclusion and outlook

In recent years, significant progress has been made in the field of neoadjuvant therapy for GC, particularly in the application of molecular biomarkers and AI technologies. Currently, a variety of molecular biomarkers related to neoadjuvant therapy for gastric cancer have been extensively studied, including the SII, miRNAs, and ASPH. These biomarkers not only provide new targets for personalized treatment of GC, but some have already entered clinical research stages and are being used for treatment decision-making and efficacy prediction. However, despite the fact that neoadjuvant therapy has improved the prognosis of LAGC patients to some extent, not all patients benefit from it, and the heterogeneity of treatment efficacy remains a major challenge in clinical practice. With the rapid development of AI technology, especially the application of deep learning and large models, breakthroughs have been made in the efficacy prediction and patient selection for neoadjuvant therapy in GC. AI can not only optimize treatment strategies by integrating multiple biomarker data, but also construct predictive models through in-depth analysis of radiomic data, thereby more accurately identifying patients who are likely to benefit from neoadjuvant therapy. This AI model based on multimodal data provides new insights for personalized decision-making in neoadjuvant therapy for gastric cancer.

In this paper, we systematically integrated blood biomarkers (e.g., SII), molecular markers (e.g., miRNA, ASPH), and imaging histological features to construct a multidimensional prediction model. Compared with traditional single-dimensional studies through cross-validation of multi-omics data, the accuracy of efficacy prediction and clinical application potential were significantly improved. For example, SII combined with miRNA dynamic monitoring can identify chemotherapy-resistant patients at an early stage, while the imaging histology model (DLDRN) further extracts tumor heterogeneity features through deep learning to support precise preoperative stratification.

Looking forward, the clinical translation of integrated biomarker and radiomic models necessitates a coordinated, multi-stage roadmap. In the short-to-mid term (1–3 years), the priority must be the prospective validation of the most promising signatures, such as dynamic SII and specific miRNA panels, within large-scale, international multi-center cohorts. This phase must be coupled with rigorous standardization of assays and radiomic feature extraction protocols to ensure reproducibility. Concurrently, in the mid-term (2–4 years), research efforts should focus on the development of AI-driven platforms capable of seamlessly integrating multi-omics data into clinically interpretable tools for point-of-care decision support. Finally, the long-term goal (3-5+ years) involves the design and execution of novel clinical trial paradigms, such as biomarker-stratified umbrella or basket trials, to prospectively demonstrate that this multi-modal guidance strategy improves patient survival and quality of life. This clear, phased roadmap provides a strategic framework for translating the promising synergy of biomarkers and radiomics into tangible benefits for gastric cancer patients worldwide.
